# Supplementary material for: Melatonin Targets Metabolism in Head and Neck Cancer Cells by Regulating Mitochondrial Structure and Function
Source: Antioxidants (Basel). 2021 Apr 14;10(4):603. doi: 10.3390/antiox10040603 (PMC8070770; doi:10.3390/antiox10040603)
Supplement: Supplementary file 1 [file antioxidants-10-00603-s001.pdf]

Supplementary Information

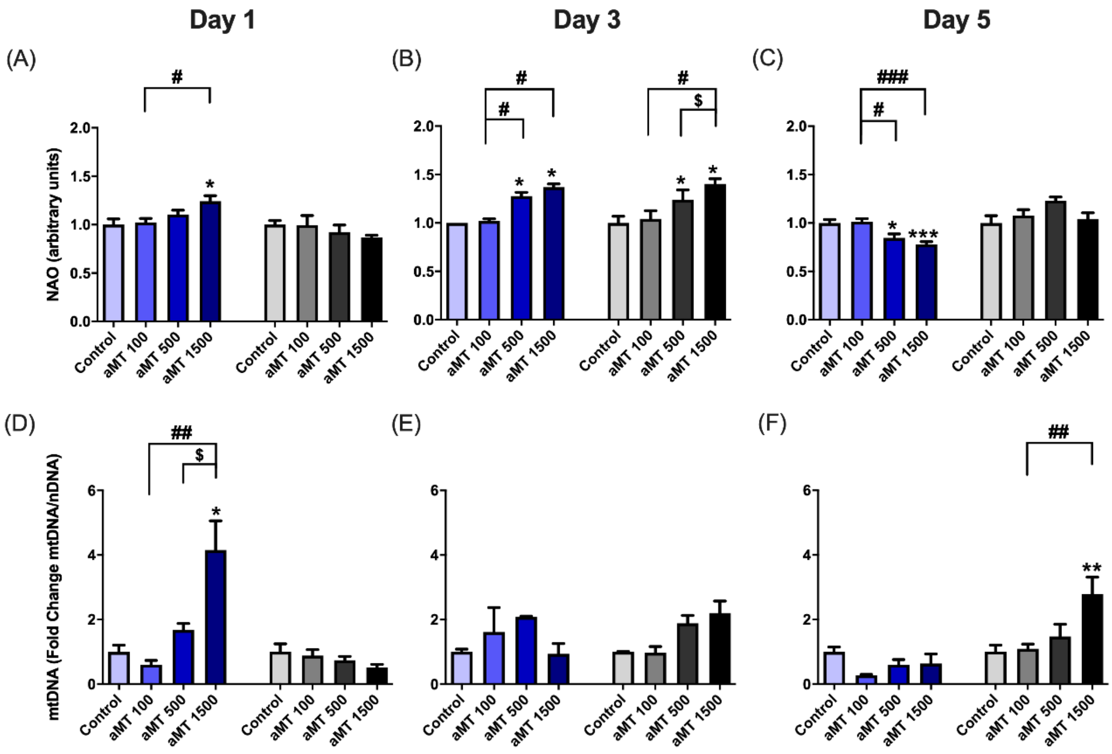

Figure S1. Melatonin-induced changes in mitochondrial morphology. Alterations in mitochondrial mass (NAO) (**A–C**) and mtDNA (**D–F**) in Cal-27 and SCC-9 cells. n = 6 per group. Data are presented as mean ± SEM. *$p < 0.05$, **$p < 0.01$, ***$p < 0.001$ vs. control; #p <0.05, ##$p < 0.01$, ###p < .001 vs. aMT 100 μM group; $\$p$ < 0.05.

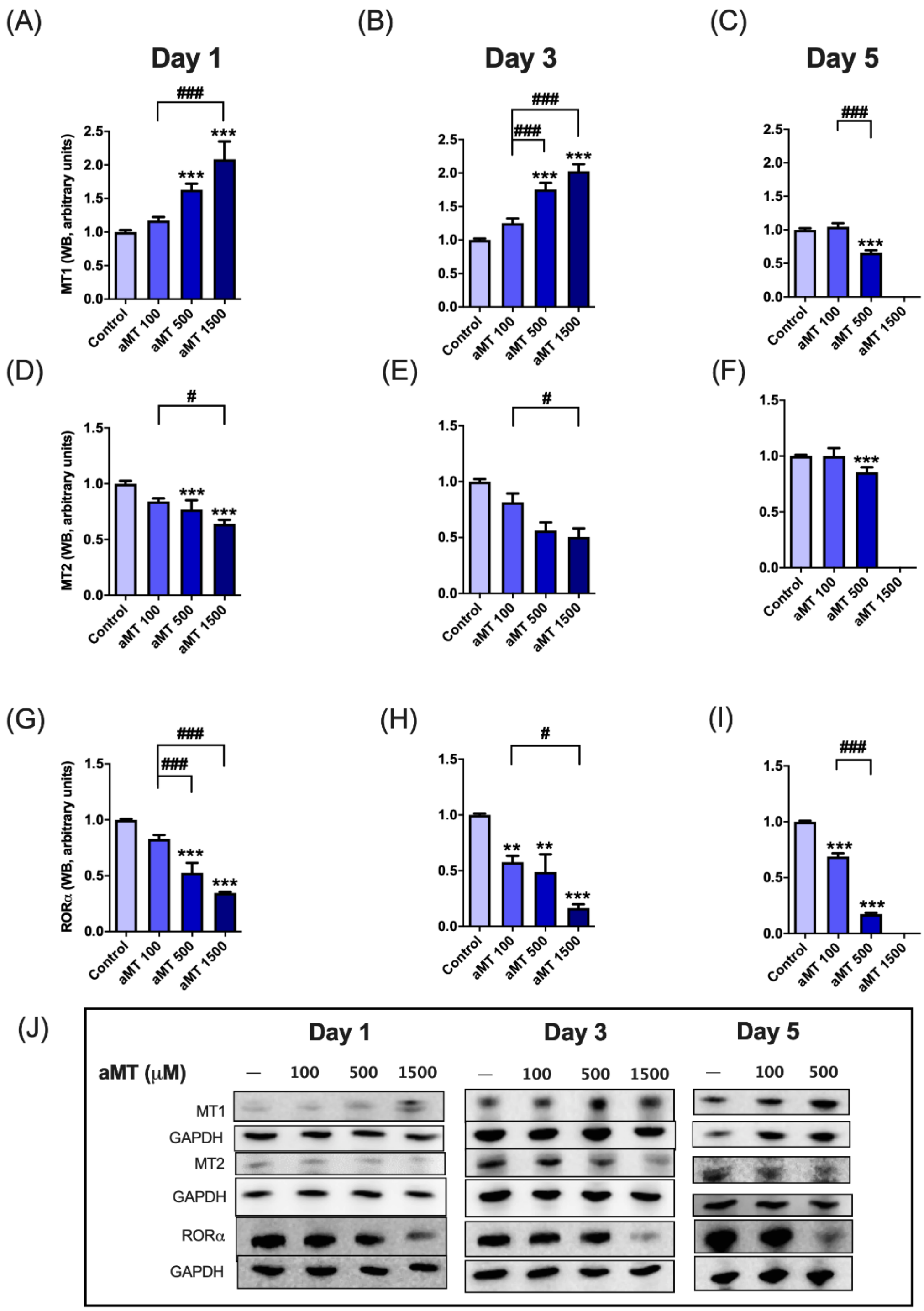

Figure S2. Modulation of melatonin receptor expression by melatonin in Cal-27 cells. Western Blot analysis of MT1 (**A**–**C**, **J**), MT2 (**D**–**F**, **J**) and RORα (**G**–**J**). n = 6 per group. Data are presented as mean ± SEM. $^{**}p < 0.01$, $^{***}p < 0.001$ vs. control; $^{\#}p < 0.05$, $^{\#\#\#}p < 0.001$ vs. aMT 100 μM group.

Full western data

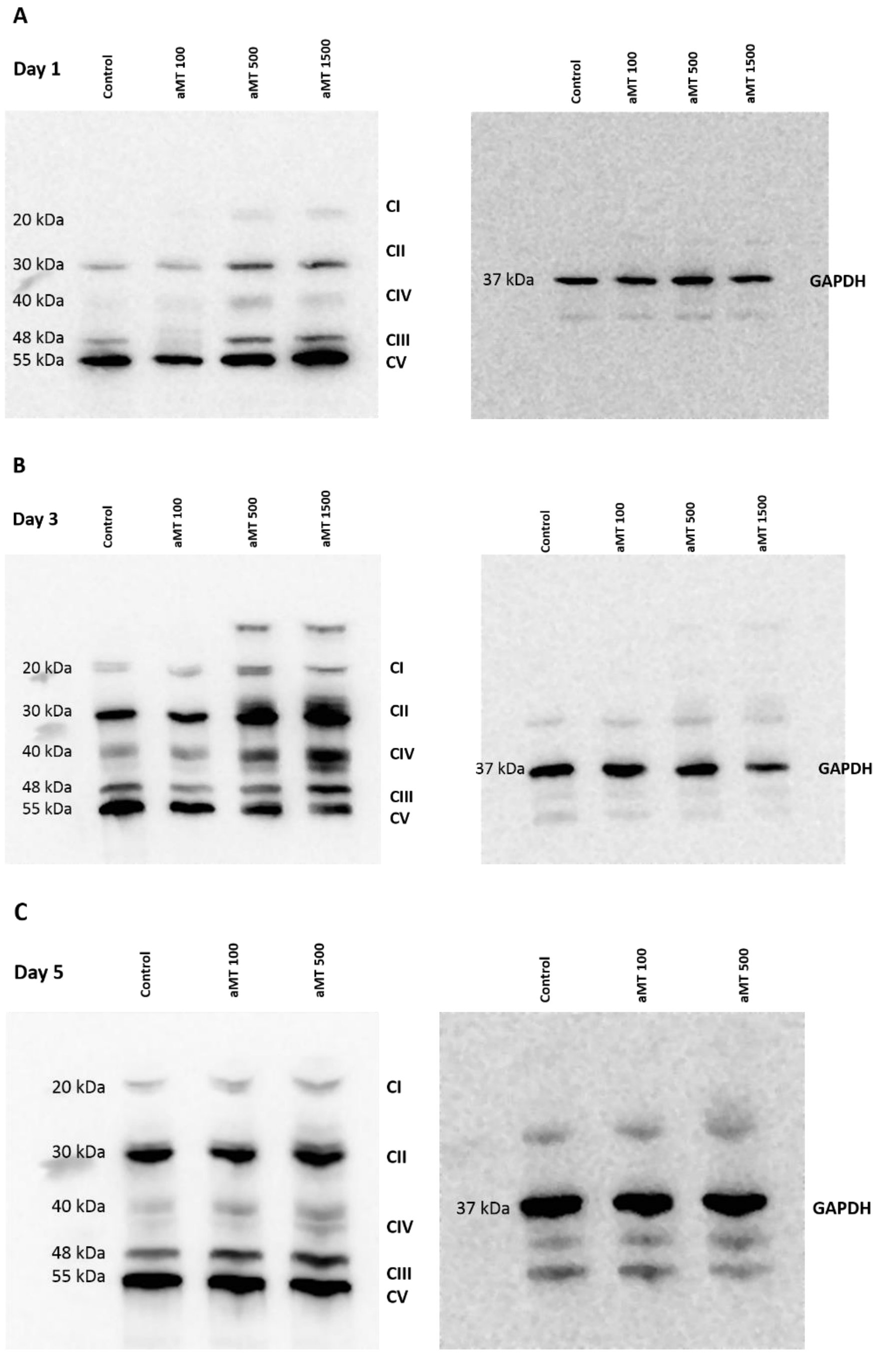

Figure S3. Full scanned Western blots shown in Figure 3. Protein content of OXPHOS proteins (Complex I-V) in figure 3 A, B (**A**); 3 C, D (**B**) and 3 E, F (**C**). Experiments were performed with Cal-27 cells in Control, aMT 100, aMT 500 and aMT 1500 groups.

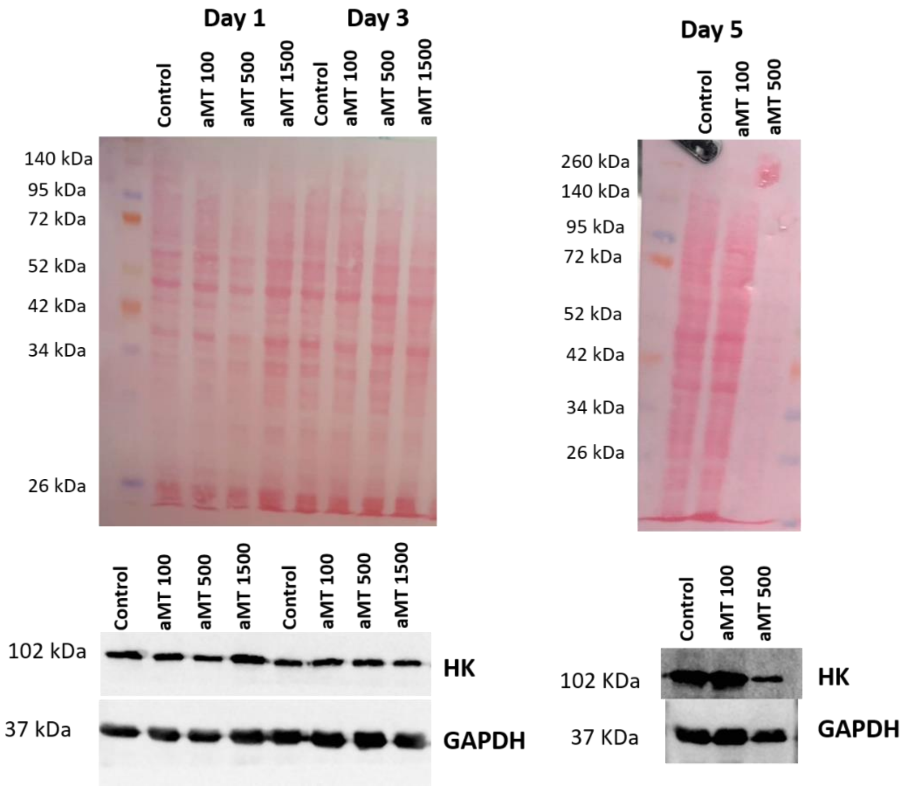

Figure S4. Full scanned Western blots shown in Figure 5 J–L. Protein content of hexokinase II in figure 5. Experiments were performed with Cal-27 cells in Control, aMT 100, aMT 500 and aMT 1500 groups.

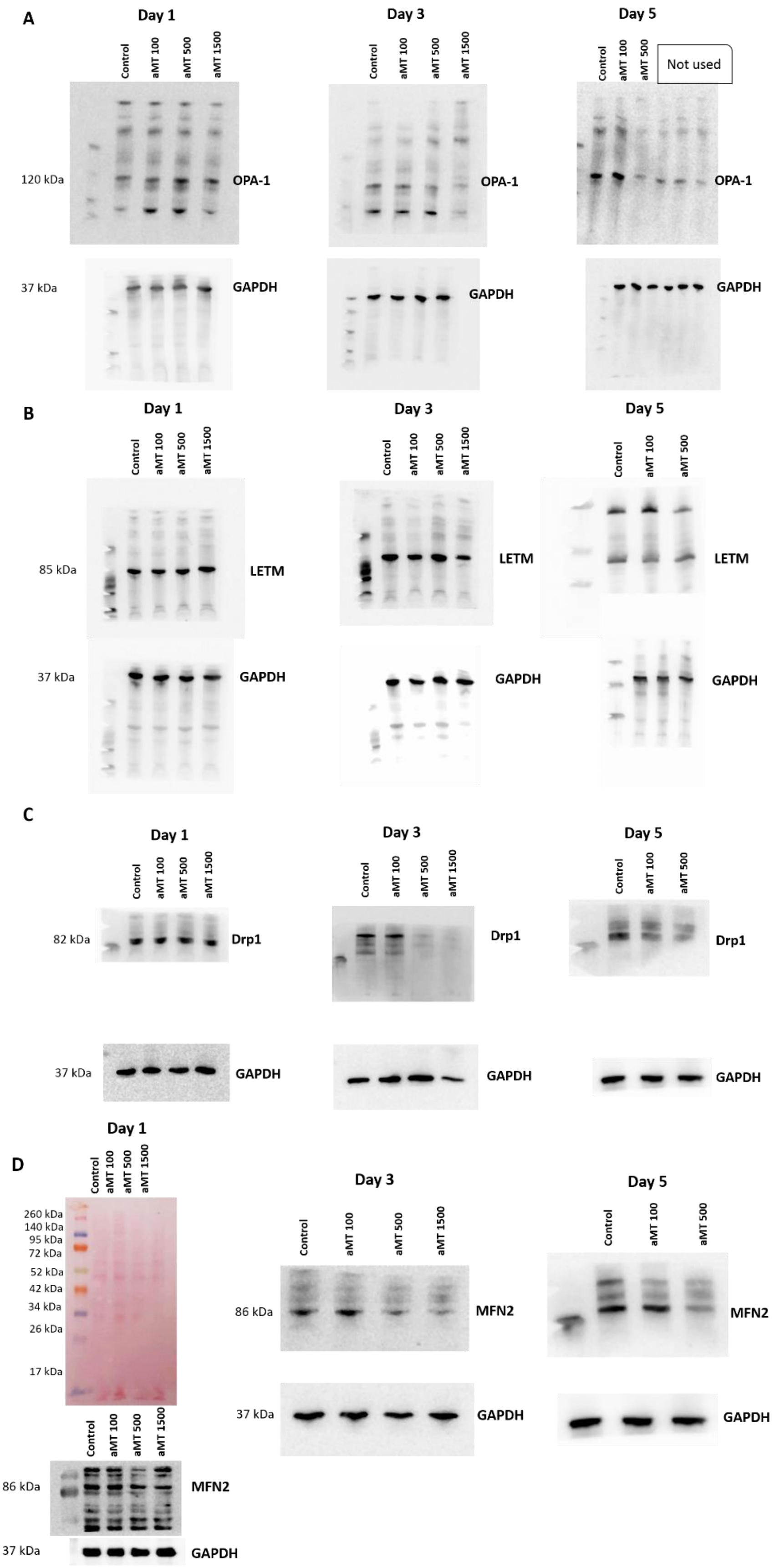

Figure S5. Full scanned Western blots shown in Figure 7. Protein content of OPA-1, LETM, MFN2 and Drp1 in Figure 7 A–C, M (**A**); D–F, M (**B**); G–I, M (**C**) and J–M (**D**). Experiments were performed with Cal-27 cells in Control, aMT 100, aMT 500 and aMT 1500 groups.

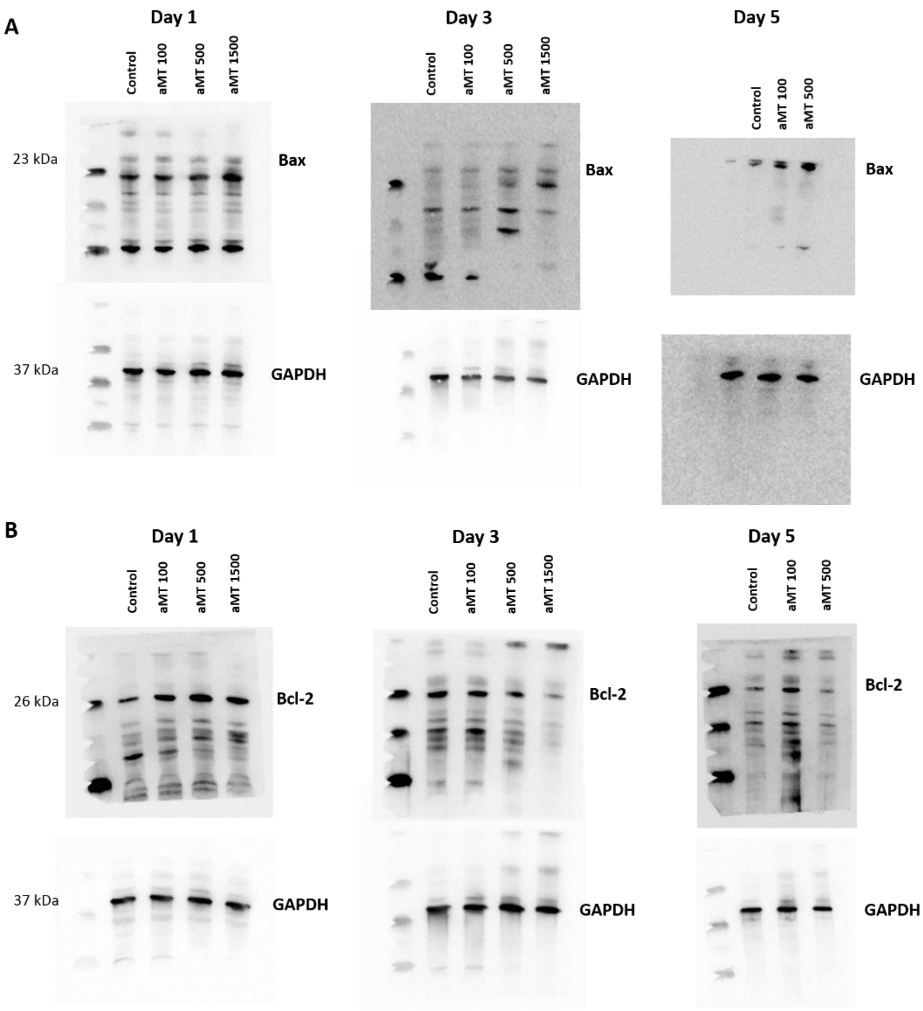

Figure S6. Full scanned Western blots shown in Figure 8 A−J. Protein content of Bax and Bcl-2 in Figure 8 A−C, J (**A**); D−F, J (**B**). Experiments were performed with Cal-27 cells in Control, aMT 100, aMT 500 and aMT 1500 groups.

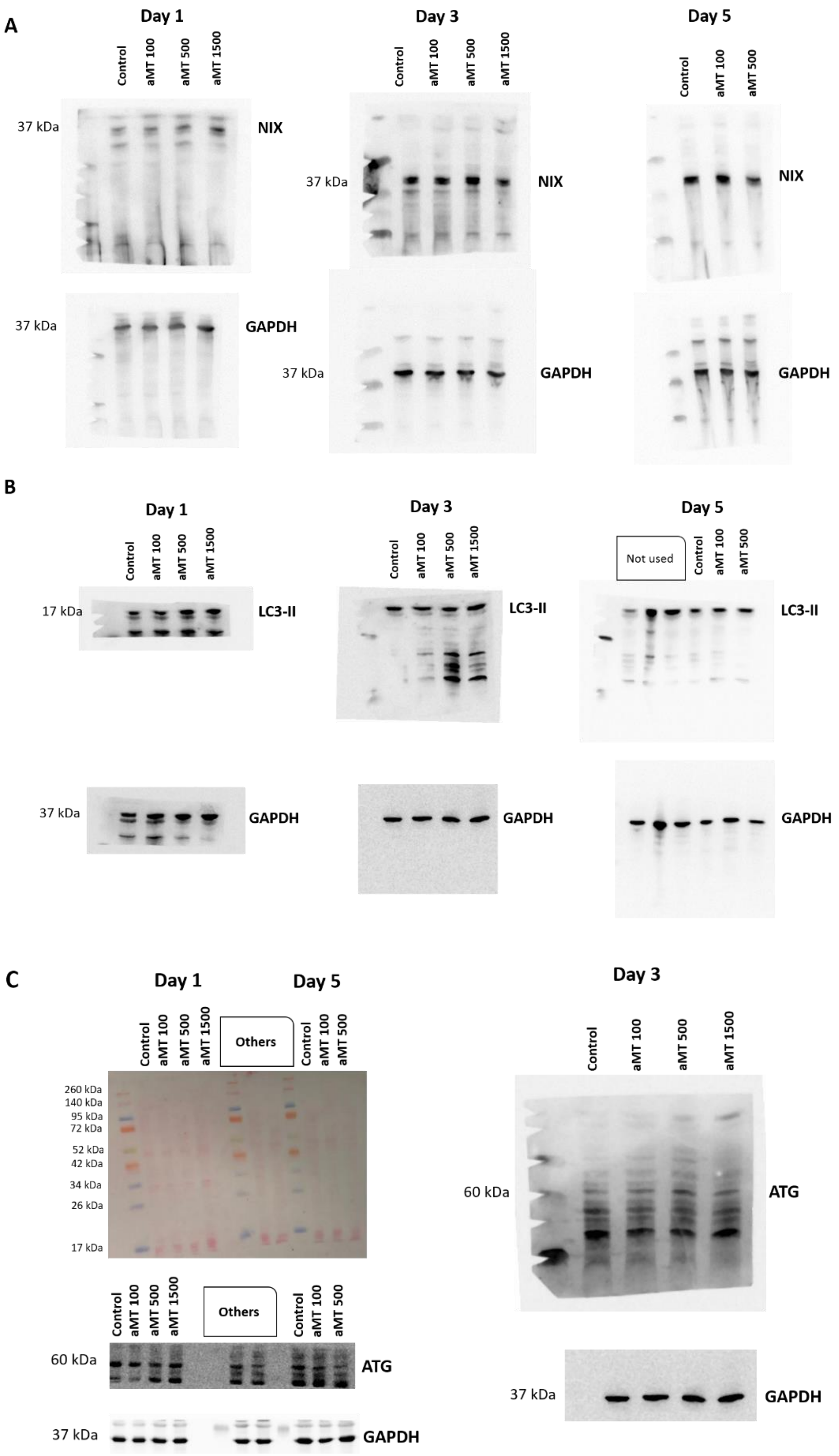

Figure S7. Full scanned Western blots shown in Figure 8 K-T. Protein content of Nix, LC3-II and ATG12-ATG5 in Figure 8 K–M, T (**A**); N–P, T (**B**) and Q–S, T (**C**). Experiments were performed with Cal-27 cells in Control, aMT 100, aMT 500 and aMT 1500 groups.

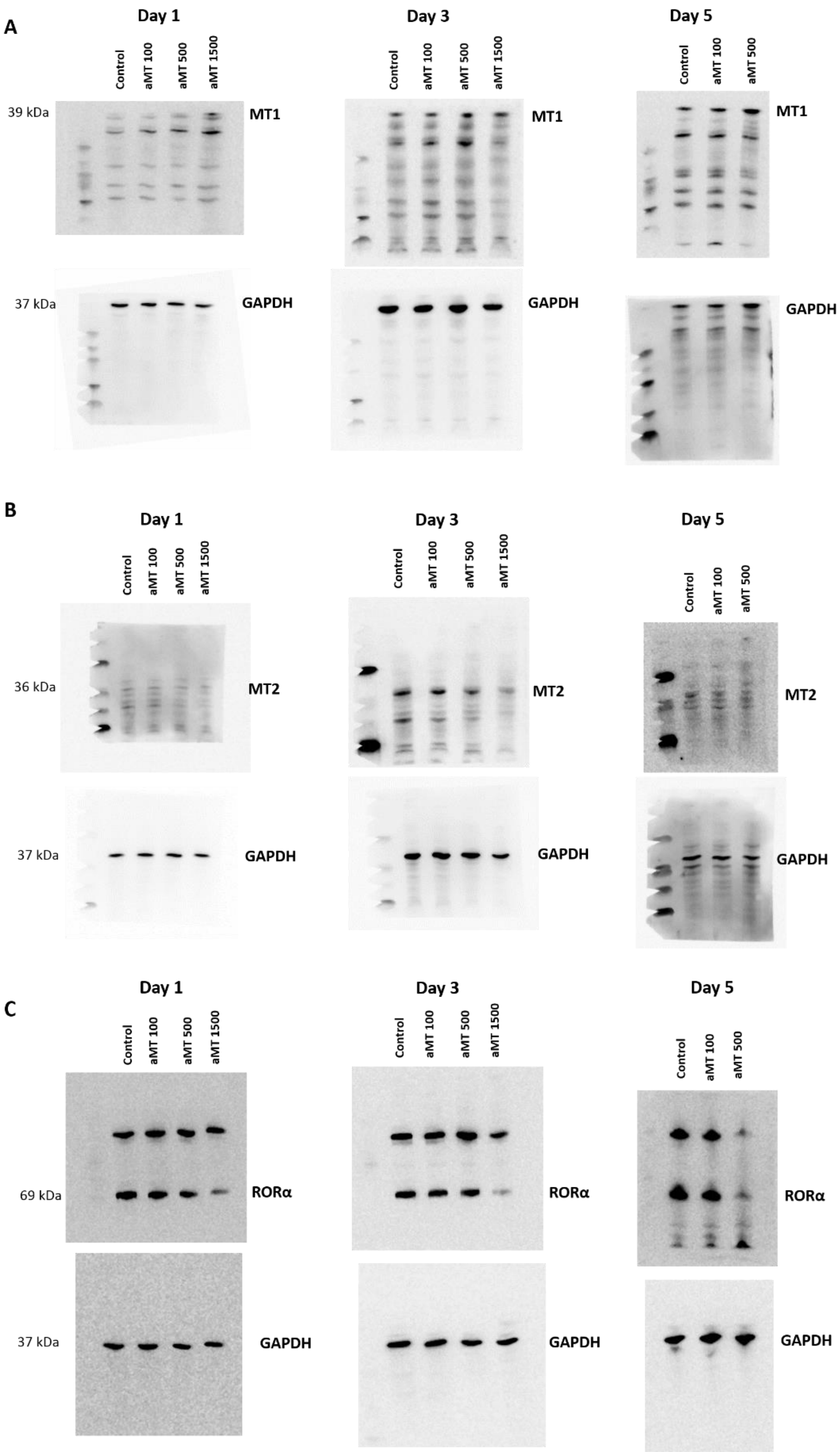

Figure S8. Full scanned Western blots shown in Figure S2. Protein content MT1, MT2 and RORα in Figure S2 A–C, J (**A**); D–F, J (**B**); K–M, T (**C**). Experiments were performed with Cal-27 cells in Control, aMT 100, aMT 500 and aMT 1500 groups.
